# Indices of Effect Existence and Significance in the Bayesian Framework

**DOI:** 10.3389/fpsyg.2019.02767

**Published:** 2019-12-10

**Authors:** Dominique Makowski, Mattan S. Ben-Shachar, S. H. Annabel Chen, Daniel Lüdecke

**Affiliations:** ^1^School of Social Sciences, Nanyang Technological University, Singapore, Singapore; ^2^Department of Psychology, Ben-Gurion University of the Negev, Beersheba, Israel; ^3^Centre for Research and Development in Learning, Nanyang Technological University, Singapore, Singapore; ^4^Lee Kong Chian School of Medicine, Nanyang Technological University, Singapore, Singapore; ^5^Department of Medical Sociology, University Medical Center Hamburg-Eppendorf, Hamburg, Germany

**Keywords:** Bayesian, significance, NHST, *p*-value, Bayes factors

## Abstract

Turmoil has engulfed psychological science. Causes and consequences of the reproducibility crisis are in dispute. With the hope of addressing some of its aspects, Bayesian methods are gaining increasing attention in psychological science. Some of their advantages, as opposed to the frequentist framework, are the ability to describe parameters in probabilistic terms and explicitly incorporate prior knowledge about them into the model. These issues are crucial in particular regarding the current debate about statistical significance. Bayesian methods are not necessarily the only remedy against incorrect interpretations or wrong conclusions, but there is an increasing agreement that they are one of the keys to avoid such fallacies. Nevertheless, its flexible nature is its power and weakness, for there is no agreement about what indices of “significance” should be computed or reported. This lack of a consensual index or guidelines, such as the frequentist *p*-value, further contributes to the unnecessary opacity that many non-familiar readers perceive in Bayesian statistics. Thus, this study describes and compares several Bayesian indices, provide intuitive visual representation of their “behavior” in relationship with common sources of variance such as sample size, magnitude of effects and also frequentist significance. The results contribute to the development of an intuitive understanding of the values that researchers report, allowing to draw sensible recommendations for Bayesian statistics description, critical for the standardization of scientific reporting.

## Introduction

The Bayesian framework is quickly gaining popularity among psychologists and neuroscientists (Andrews and Baguley, 2013), for reasons such as flexibility, better accuracy in noisy data and small samples, less proneness to type I errors, the possibility of introducing prior knowledge into the analysis and the intuitiveness and straightforward interpretation of results (Kruschke, 2010; Kruschke et al., 2012; Etz and Vandekerckhove, 2016; Wagenmakers et al., 2016, 2018; Dienes and Mclatchie, 2018). On the other hand, the frequentist approach has been associated with the focus on *p*-values and null hypothesis significance testing (NHST). The misinterpretation and misuse of *p*-values, so called “p-hacking” (Simmons et al., 2011), has been shown to critically contribute to the reproducibility crisis in psychological science (Chambers et al., 2014; Szucs and Ioannidis, 2016). The reliance on *p*-values has been criticized for its association with inappropriate inference, and effects can be drastically overestimated, sometimes even in the wrong direction, when estimation is tied to statistical significance in highly variable data (Gelman, 2018). Power calculations allow researchers to control the probability of falsely rejecting the null hypothesis, but do not completely solve this problem. For instance, the “false-alarm probability” of even very small *p*-values can be much higher than expected (Nuzzo, 2014). In response, there is an increasing belief that the generalization and utilization of the Bayesian framework is one way of overcoming these issues (Maxwell et al., 2015; Etz and Vandekerckhove, 2016; Marasini et al., 2016; Wagenmakers et al., 2017; Benjamin et al., 2018; Halsey, 2019).

The tenacity and resilience of the *p*-value as an index of significance is remarkable, despite the long-lasting criticism and discussion about its misuse and misinterpretation (Gardner and Altman, 1986; Cohen, 1994; Anderson et al., 2000; Fidler et al., 2004; Finch et al., 2004). This endurance might be informative on how such indices, and the accompanying heuristics applied to interpret them (e.g., assigning thresholds like 0.05, 0.01, and 0.001 to certain levels of significance), are useful and necessary for researchers to gain an intuitive (although possibly simplified) understanding of the interactions and structure of their data. Moreover, the utility of such an index is most salient in contexts where decisions must be made and rationalized (e.g., in medical settings). Unfortunately, these heuristics can become severely rigidified, and meeting significance has become a goal unto itself rather than a tool for understanding the data (Cohen, 1994; Kirk, 1996). This is particularly problematic given that *p*-values can only be used to reject the null hypothesis and not to accept it as true, because a statistically non-significant result does not mean that there is no difference between groups or no effect of a treatment (Wagenmakers, 2007; Amrhein et al., 2019).

While significance testing (and its inherent categorical interpretation heuristics) might have its place as a complementary perspective to effect estimation, it does not preclude the fact that improvements are needed. For instance, one possible advance could focus on improving the understanding of the values being used, for instance, through a new, simpler, index. Bayesian inference allows making intuitive probability statements of an effect, as opposed to the less straightforward mathematical definition of the *p*-value, that contributes to its common misinterpretation. Another improvement could be found in providing an intuitive understanding (e.g., by visual means) of the behavior of the indices in relationship with main sources of variance, such as sample size, noise, or effect presence. Such better overall understanding of the indices would hopefully act as a barrier against their mindless reporting by allowing the users to nuance the interpretations and conclusions that they draw.

The Bayesian framework offers several alternative indices for the *p*-value. To better understand these indices, it is important to point out one of the core differences between Bayesian and frequentist methods. From a frequentist perspective, the effects are fixed (but unknown) and data are random. On the other hand, instead of having single estimates of some “true effect” (for instance, the “true” correlation between *x* and *y*), Bayesian methods compute the probability of different effects values *given* the observed data (and some prior expectation), resulting in a distribution of possible values for the parameters, called the posterior distribution. The description of the posterior distribution (e.g., through its centrality, dispersion, etc.) allows to draw conclusions from Bayesian analyses.

Bayesian “significance” testing indices could be roughly grouped into three overlapping categories: Bayes factors, posterior indices and Region of Practical Equivalence (ROPE)-based indices. Bayes factors are a family of indices of relative evidence of one model over another (e.g., the null vs. the alternative hypothesis; Jeffreys, 1998; Ly et al., 2016). Aside from having a straightforward interpretation (“given the observed data, is the null hypothesis of an absence of an effect more, or less likely?”), they allow to quantify the evidence in favor of the null hypothesis (Dienes, 2014; Jarosz and Wiley, 2014). However, its use for parameters description in complex models is still a matter of debate (Wagenmakers et al., 2010; Heck, 2019), being highly dependent on the specification of priors (Etz et al., 2018; Kruschke and Liddell, 2018). On the contrary, “posterior indices” reflect objective characteristics of the posterior distribution, for instance the proportion of strictly positive values. They also allow to derive legitimate statements that indicate the probability of an effect falling in a given range similar to the misleading conclusions related to frequentist confidence intervals. Finally, ROPE-based indices are related to the redefinition of the null hypothesis from the classic point-null hypothesis to a range of values considered negligible or too small to be of any practical relevance (the Region of Practical Equivalence – ROPE; Kruschke, 2014; Lakens, 2017; Lakens et al., 2018), usually spread equally around 0 (e.g., [−0.1; 0.1]). The idea behind this index is that an effect is almost never exactly zero, but instead can be very tiny, with no practical relevance. It is interesting to note that this perspective unites significance testing with the focus on effect size (involving a discrete separation between at least two categories: negligible and non-negligible), which finds an echo in recent statistical recommendations (Ellis and Steyn, 2003; Sullivan and Feinn, 2012; Simonsohn et al., 2014).

Despite the richness provided by the Bayesian framework and the availability of multiple indices, no consensus has yet emerged on which ones to be used. Literature continues to bloom in a raging debate, often polarized between proponents of the Bayes factor as the supreme index and its detractors (Spanos, 2013; Robert, 2014, 2016; Wagenmakers et al., 2019), with strong theoretical arguments being developed on both sides. Yet no practical, empirical and direct comparison between these indices has been done. This might be a deterrent for scientists interested in adopting the Bayesian framework. Moreover, this gray area can increase the difficulty of readers or reviewers unfamiliar with the Bayesian framework to follow the assumptions and conclusions, which could in turn generate unnecessary doubt upon an entire study. While we think that such indices of significance and their interpretation guidelines (in the form of rules of thumb) are useful in practice, we also strongly believe that they should be accompanied with the understanding of their “behavior” in relationship with major sources of variance, such as sample size, noise or effect presence. This knowledge is important for people to implicitly and intuitively appraise the meaning and implication of the mathematical values they report. Such an understanding could prevent the crystallization of the possible heuristics and categories derived from such indices, as has unfortunately occurred for the *p*-values.

Thus, based on the simulation of linear and logistic regressions (arguably some of the most widely used models in the psychological sciences), the present work aims at comparing several indices of effect “significance,” provide visual representations of the “behavior” of such indices in relationship with sample size, noise and effect presence, as well as their relationship to frequentist *p*-values (an index which, beyond its many flaws, is well known and could be used as a reference for Bayesian neophytes), and finally draw recommendations for Bayesian statistics reporting.

## Materials and Methods

### Data Simulation

We simulated datasets suited for linear and logistic regression and started by simulating an independent, normally distributed *x* variable (with mean 0 and SD 1) of a given sample size. Then, the corresponding *y* variable was added, having a perfect correlation (in the case of data for linear regressions) or as a binary variable perfectly separated by *x*. The case of no effect was simulated by creating a *y* variable that was independent of (i.e., not correlated to) *x*. Finally, a Gaussian noise (the error) was added to the x variable before its standardization, which in turn decreases the standardized coefficient (the effect size).

The simulation aimed at modulating the following characteristics: *outcome type* (linear or logistic regression), *sample size* (from 20 to 100 by steps of 10), *null hypothesis* (original regression coefficient from which data is drawn prior to noise addition, 1 – presence of “true” effect, or 0 – absence of “true” effect) and *noise* (Gaussian noise applied to the predictor with SD uniformly spread between 0.666 and 6.66, with 1000 different values), which is directly related to the absolute value of the coefficient (i.e., the effect size). We generated a dataset for each combination of these characteristics, resulting in a total of 36,000 (2 model types × 2 presence/absence of effect × 9 sample sizes × 1,000 noise variations) datasets. The code used for data generation is available on GitHub^1^. Note that it takes usually several days/weeks for the generation to complete.

### Indices

**FIGURE 1:**
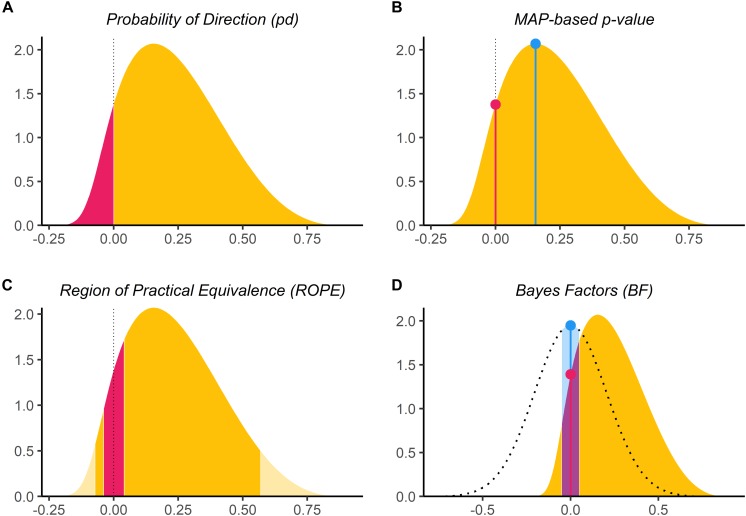
Bayesian indices of effect existence and significance. **(A)** The probability of Direction (*pd*) is defined as the proportion of the posterior distribution that is of the median’s sign (the size of the yellow area relative to the whole distribution). **(B)** The MAP-based *p*-value is defined as the density value at 0 – the height of the red lollipop, divided by the density at the Maximum *A Posteriori* (MAP) – the height of the blue lollipop. **(C)** The percentage in ROPE corresponds to the red area relative to the distribution [with or without tails for ROPE (*full*) and ROPE (95%), respectively]. **(D)** The Bayes factor (vs. 0) corresponds to the point-null density of the prior (the blue lollipop on the dotted distribution) divided by that of the posterior (the red lollipop on the yellow distribution), and the Bayes factor (vs. ROPE) is calculated as the odds of the prior falling within vs. outside the ROPE (the blue area on the dotted distribution) divided by that of the posterior (the red area on the yellow distribution).

For each of these datasets, Bayesian and frequentist regressions were fitted to predict *y* from *x* as a single unique predictor. We then computed the following seven indices from all simulated models (see Figure 1), related to the effect of *x*.

#### Frequentist *p*-Value

This was the only index computed by the frequentist version of the regression. The *p*-value represents the probability that for a given statistical model, when the null hypothesis is true, the effect would be greater than or equal to the observed coefficient (Wasserstein and Lazar, 2016).

#### Probability of Direction (*pd*)

The *Probability of Direction (pd)* varies between 50 and 100% and can be interpreted as the probability that a parameter (described by its posterior distribution) is strictly positive or negative (whichever is the most probable). It is mathematically defined as the proportion of the posterior distribution that is of the median’s sign (Makowski et al., 2019).

#### MAP-Based *p*-Value

The *MAP-based p-value* is related to the odds that a parameter has against the null hypothesis (Mills and Parent, 2014; Mills, 2017). It is mathematically defined as the density value at 0 divided by the density at the Maximum *A Posteriori* (MAP), i.e., the equivalent of the mode for continuous distributions.

#### ROPE (95%)

The *ROPE* (95%) refers to the percentage of the 95% Highest Density Interval (HDI) that lies within the ROPE. As suggested by Kruschke (2014), the Region of Practical Equivalence (ROPE) was defined as range from −0.1 to 0.1 for linear regressions and its equivalent, −0.18 to 0.18, for logistic models (based on the $\pi /\sqrt{3}$ formula to convert log odds ratios to standardized differences; Cohen, 1988). Although we present the “95% percentage” because of the history of this index and of its widespread use, the reader should note that this value was recently challenged due to its arbitrary nature (McElreath, 2018).

#### ROPE (Full)

The *ROPE (full)* is similar to *ROPE (95%)*, with the exception that it refers to the percentage of the *whole* posterior distribution that lies within the ROPE.

#### Bayes Factor (vs. 0)

The Bayes Factor (*BF*) used here is based on prior and posterior distributions of a single parameter. In this context, the Bayes factor indicates the degree by which the mass of the posterior distribution has shifted further away from or closer to the null value (0), relative to the prior distribution, thus indicating if the null hypothesis has become less or more likely given the observed data. The *BF* was computed as a Savage-Dickey density ratio, which is also an approximation of a Bayes factor comparing the marginal likelihoods of the model against a model in which the tested parameter has been restricted to the point-null (Wagenmakers et al., 2010).

#### Bayes Factor (vs. ROPE)

The *Bayes factor* (vs. *ROPE*) is similar to the *Bayes factor* (vs. 0), but instead of a point-null, the null hypothesis is a range of negligible values (defined here same as for the ROPE indices). The *BF* was computed by comparing the prior and posterior odds of the parameter falling within vs. outside the ROPE (see *Non-overlapping Hypotheses* in Morey and Rouder, 2011). This measure is closely related to the *ROPE (full)*, as it can be formally defined as the ratio between the *ROPE (full)* odds for the posterior distribution and the *ROPE (full)* odds for the prior distribution:

$${\text{BF}}_{\text{ROPE}}=\frac{\mathrm{odds}\,\left({\mathrm{ROPE}}_{\mathrm{full}\,\mathrm{posterior}}\right)}{\mathrm{odds}\,\left({\mathrm{ROPE}}_{\mathrm{full}\,\mathrm{prior}}\right)}$$

### Data Analysis

In order to achieve the two-fold aim of this study; (1) comparing Bayesian indices and (2) provide visual guides for an intuitive understanding of the numeric values in relation to a known frame of reference (the frequentist *p*-value), we will start by presenting the relationship between these indices and main sources of variance, such as sample size, noise and null hypothesis (true if absence of effect, false if presence of effect). We will then compare Bayesian indices with the frequentist *p*-value and its commonly used thresholds (0.05, 0.01, 0.001). Finally, we will show the mutual relationship between three recommended Bayesian candidates. Taken together, these results will help us outline guides to ease the reporting and interpretation of the indices.

In order to provide an intuitive understanding of values, data processing will focus on creating clear visual figures to help the user grasp the patterns and variability that exists when computing the investigated indices. Nevertheless, we decided to also mathematically test our claims in cases where the graphical representation begged for a deeper investigation. Thus, we fitted two regression models to assess the impact of sample size and noise, respectively. For these models (but not for the figures), to ensure that any differences between the indices are not due to differences in their scale or distribution, we converted all indices to the same scale by normalizing the indices between 0 and 1 (note that *BF*s were transformed to posterior probabilities, assuming uniform prior odds) and reversing the *p*-values, the MAP-based *p*-values and the ROPE indices so that a higher value corresponds to stronger “significance.”

The statistical analyses were conducted using R (R Core Team, 2019). Computations of Bayesian models were done using the *rstanarm* package (Goodrich et al., 2019), a wrapper for Stan probabilistic language (Carpenter et al., 2017). We used Markov Chain Monte Carlo sampling (in particular, Hamiltonian Monte Carlo; Gelman et al., 2014) with 4 chains of 2000 iterations, half of which used for warm-up. Mildly informative priors (a normal distribution with mean 0 and SD 1) were used for the parameter in all models. The Bayesian indices were calculated using the *bayestestR* package (Makowski et al., 2019).

## Results

### Impact of Sample Size

**FIGURE 2:**
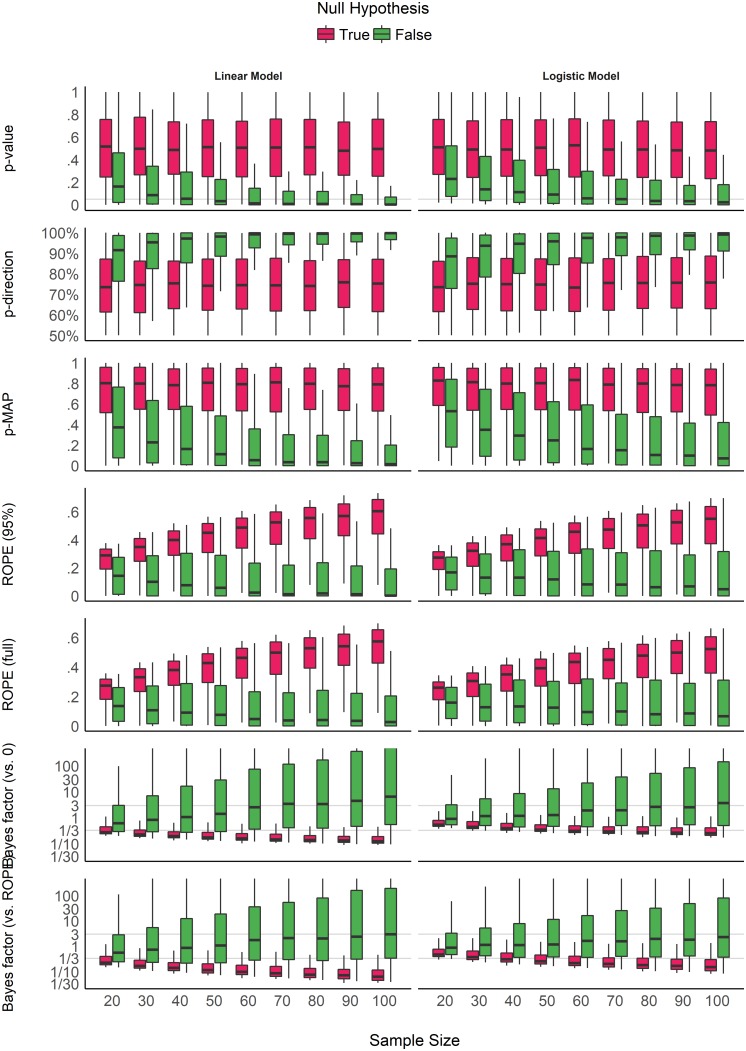
Impact of sample size on the different indices, for linear and logistic models, and when the null hypothesis is true or false. Gray vertical lines for *p*-values and Bayes factors represent commonly used thresholds.

Figure 2 shows the sensitivity of the indices to sample size. The *p*-value, the *pd* and the MAP-based *p*-value are sensitive to sample size only in case of the presence of a true effect (when the null hypothesis is false). When the null hypothesis is true, all three indices are unaffected by sample size. In other words, these indices reflect the amount of observed evidence (the sample size) for the presence of an effect (i.e., against the null hypothesis being true), but not for the absence of an effect. The *ROPE* indices, however, appear as strongly modulated by the sample size when there is no effect, suggesting their sensitivity to the amount of evidence for the absence of effect. Finally, the figure suggests that *BFs* are sensitive to sample size for both presence and absence of true effect.

Consistently with Figure 2 and Table 1, the model investigating the sensitivity of sample size on the different indices suggests that *BF* indices are sensitive to sample size both when an effect is present (null hypothesis is false) and absent (null hypothesis is true). *ROPE* indices are particularly sensitive to sample size when the null hypothesis is true, while *p*-value, *pd* and MAP-based *p*-value are only sensitive to sample size when the null hypothesis is false, in which case they are more sensitive than *ROPE* indices. These findings can be related to the concept of consistency: as the number of data points increases, the statistic converges toward some “true” value. Here, we observe that *p*-value, *pd* and the MAP-based *p*-value are consistent only when the null hypothesis is false. In other words, as sample size increases, they tend to reflect more strongly that the effect is present. On the other hand, *ROPE* indices appear as consistent when the effect is absent. Finally, *BFs* are consistent both when the effect is absent and when it is present, and *BF* (vs. *ROPE*), compared to *BF* (vs. 0), is more sensitive to sample size when the null hypothesis is true, and *ROPE (full)* is overall slightly more consistent than *ROPE* (95%).

**TABLE 1:** Sensitivity to sample size.

**Index**	**Linear models/presence of effect**	**Linear models/absence of effect**	**Logistic models/presence of effect**	**Logistic models/absence of effect**
*p*-value	0.166	0.008	0.157	0.020
*p*-direction	0.171	0.013	0.154	0.024
*p*-MAP	0.239	0.002	0.238	0.032
ROPE (95%)	0.033	0.359	0.008	0.310
ROPE (full)	0.025	0.363	0.016	0.315
Bayes factor (vs. 0)	0.198	0.116	0.116	0.141
Bayes factor (vs. ROPE)	0.152	0.136	0.078	0.180

*This table shows the standardized coefficient between the sample size and the value of each index, adjusted for error, and stratified by model type and presence of true effect. The stronger the coefficient is, the stronger the relationship with sample size.*

### Impact of Noise

**FIGURE 3:**
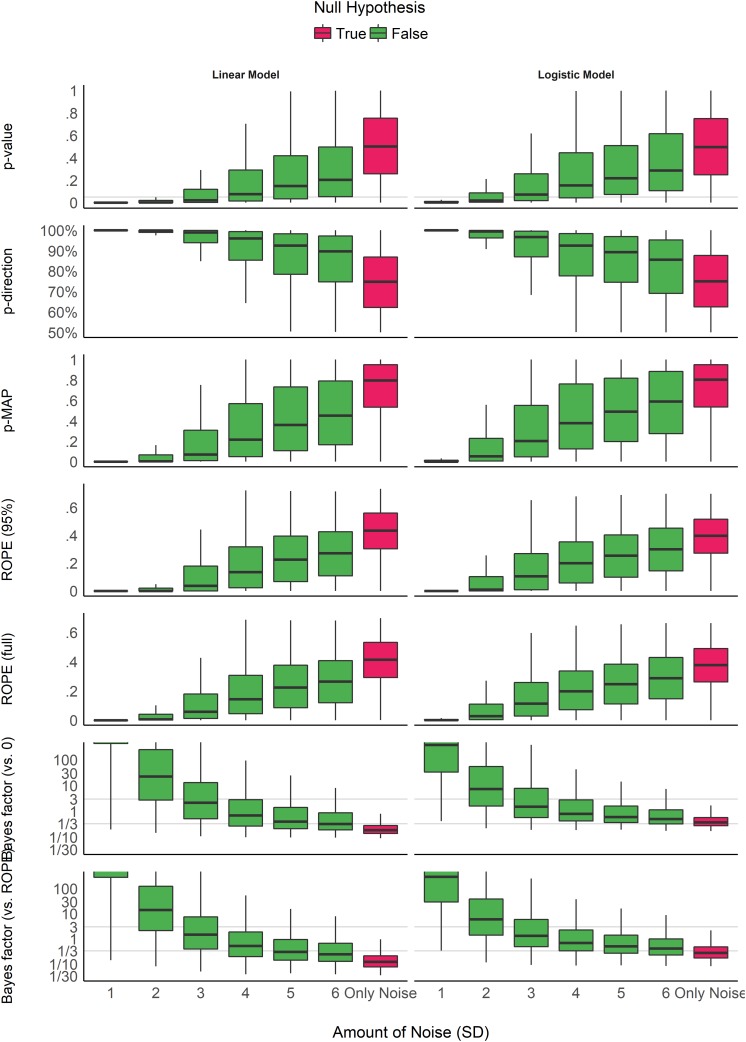
Impact of noise. The noise corresponds to the standard deviation of the Gaussian noise that was added to the generated data. It is related to the magnitude of the parameter (the more noise there is, the smaller the coefficient). Gray vertical lines for *p*-values and Bayes factors represent commonly used thresholds. The scale is capped for the Bayes factors as these extend to infinity.

Figure 3 shows the indices’ sensitivity to noise. Unlike the patterns of sensitivity to sample size, the indices display more similar patterns in their sensitivity to noise (or magnitude of effect). All indices are unidirectional impacted by noise: as noise increases, the observed coefficients decrease in magnitude, and the indices become less “pronounced” (respectively to their direction). However, it is interesting to note that the variability of the indices seems differently impacted by noise. For the *p*-values, the *pd* and the ROPE indices, the variability increases as the noise increases. In other words, small variation in small observed coefficients can yield very different values. On the contrary, the variability of BFs decreases as the true effect tends toward 0. For the MAP-based *p*-value, the variability appears to be the highest for moderate amount of noise. This behavior seems consistent across model types.

Consistently with Figure 3 and Table 2, the model investigating the sensitivity of noise when an effect is present (as there is only noise in the absence of effect), adjusted for sample size, suggests that BFs (especially vs. ROPE), followed by the MAP-based *p*-value and percentages in *ROPE*, are the most sensitive to noise. As noise is a proxy of effect size (linearly related to the absolute value of the coefficient of the parameter), this result highlights the fact that these indices are sensitive to the magnitude of the effect. For example, as noise increases, evidence for an effect becomes weak, and data seems to support the absence of an effect (or at the very least the presence of a negligible effect), which is reflected in *BF*s being consistently smaller than 1. On the other hand, as the *p*-value and the *pd* quantify evidence only for the presence of an effect, as noise increases, they are become more dependent on larger sample size to be able to detect the presence of an effect.

**TABLE 2:** Sensitivity to noise.

**Index**	**Linear models/presence of effect**	**Logistic models/presence of effect**
*p*-value	0.35	0.40
*p*-direction	0.36	0.40
*p*-MAP	0.55	0.60
ROPE (95%)	0.45	0.45
ROPE (full)	0.46	0.45
Bayes factor (vs. 0)	0.79	0.65
Bayes factor (vs. ROPE)	0.81	0.67

*This table shows the standardized coefficient between noise and the value of each index when the true effect is present, adjusted for sample size and stratified by model type. The stronger the coefficient is, the stronger the relationship with noise.*

### Relationship With the Frequentist *p*-Value

**FIGURE 4:**
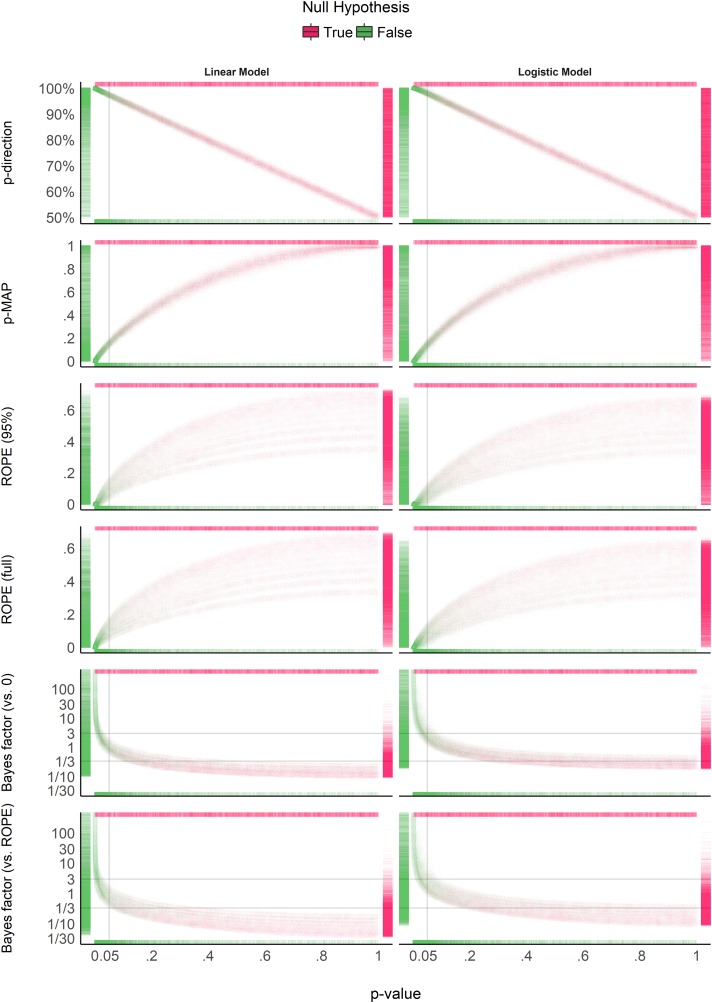
Relationship with the frequentist *p*-value. In each plot, the *p*-value densities are visualized by the marginal **top** (absence of true effect) and **bottom** (presence of true effect) markers, whereas on the **left** (presence of true effect) and **right** (absence of true effect), the markers represent the density of the index of interest. Different point shapes, representing different sample sizes, specifically illustrate its impact on the percentages in ROPE, for which each “curve line” is associated with one sample size (the bigger the sample size, the higher the percentage in ROPE).

Figure 4 suggests that the *pd* has a 1:1 correspondence with the frequentist *p*-value (through the formula *p*_two−sided_ = 2×(1−*p*_*d*_)). *BF* indices still appear as having a severely non-linear relationship with the frequentist index, mostly due to the fact that smaller *p*-values correspond to stronger evidence in favor of the presence of an effect, but the reverse is not true. *ROPE*-based percentages appear to be only weakly related to *p*-values. Critically, their relationship seems to be strongly dependent on sample size.

**FIGURE 5:**
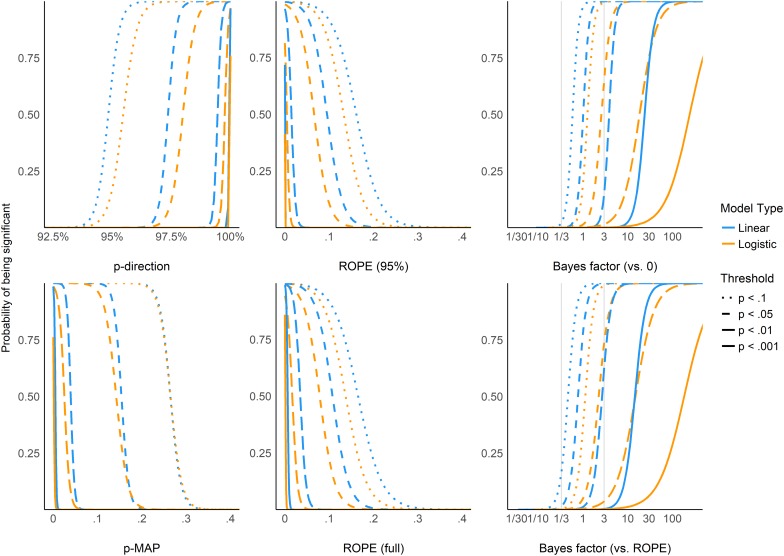
The probability of reaching different *p*-value based significance thresholds (0.1, 0.05, 0.01, 0.001 for solid, long-dashed, short-dashed, and dotted lines, respectively) for different values of the corresponding Bayesian indices.

Figure 5 shows equivalence between *p*-value thresholds (0.1, 0.05, 0.01, 0.001) and the Bayesian indices. As expected, the *pd* has the sharpest thresholds (95, 97.5, 99.5, and 99.95%, respectively). For logistic models, these threshold points appear as more conservative (i.e., Bayesian indices have to be more “pronounced” to reach the same level of significance). This sensitivity to model type is the strongest for BFs (which is possibly related to the difference in the prior specification for these two types of models).

### Relationship Between ROPE (Full), *pd*, and *BF* (vs. ROPE)

**FIGURE 6:**
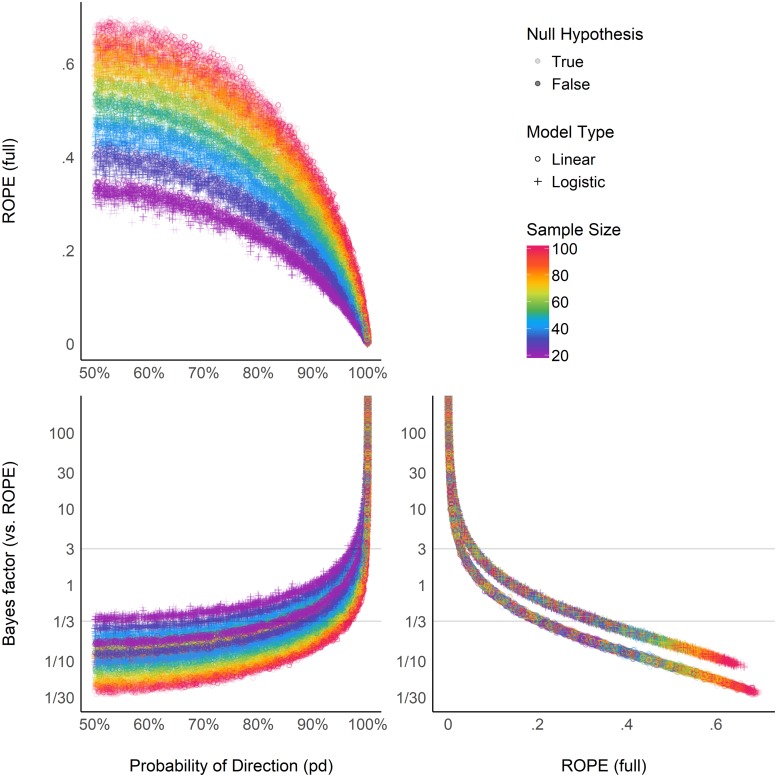
Relationship between three Bayesian indices: the probability of direction (*pd*), the percentage of the full posterior distribution in the ROPE, and the Bayes factor (vs. ROPE).

Figure 6 suggests that the relationship between the *ROPE (full)* and the *pd* might be strongly affected by the sample size, and subject to differences across model types. This seems to echo the relationship between *ROPE (full)* and *p*-value, the latter having a 1:1 correspondence with *pd*. On the other hand, the *ROPE (full)* and the *BF* (vs. *ROPE*) seem very closely related within the same model type, reflecting their formal relationship [see definition of *BF* (vs. *ROPE*) above]. Overall, these results help to demonstrate *ROPE (full)* and *BF (vs. ROPE)*’s consistency both in case of presence and absence of a true effect, whereas the *pd*, being equivalent to the *p*-value, is only consistent when the true effect is absent.

## Discussion

Based on the simulation of linear and logistic models, the present work aimed to compare several Bayesian indices of effect “significance” (see Table 3), providing visual representations of the “behavior” of such indices in relationship with important sources of variance such as sample size, noise and effect presence, as well as comparing them with the well-known and widely used frequentist *p*-value.

**TABLE 3:** Summary of Bayesian indices of effect existence and significance.

**Index**	**Interpretation**	**Definition**	**Strengths**	**Limitations**
Probability of Direction (pd)	Probability that an effect is of the same sign as the median’s	Proportion of the posterior distribution of the same sign than the median’s	Straightforward computation and interpretation. Objective property of the posterior distribution. 1:1 correspondence with the frequentist *p*-value	Limited information favoring the null hypothesis
MAP-based *p*-value	Relative odds of the presence of an effect against 0	Density value at 0 divided by the density value at the mode of the posterior distribution	Straightforward computation. Objective property of the posterior distribution	Limited information favoring the null hypothesis. Relates on density approximation. Indirect relationship between mathematical definition and interpretation
ROPE (95%)	Probability that the credible effect values are not negligible	Proportion of the 95% CI inside of a range of values defined as the ROPE	Provides information related to the practical relevance of the effects	A ROPE range needs to be arbitrarily defined. Sensitive to the scale (the unit) of the predictors. Not sensitive to highly significant effects
ROPE (full)	Probability that the effect possible values are not negligible	Proportion of the posterior distribution inside of a range of values defined as the ROPE	Provides information related to the practical relevance of the effects	A ROPE range needs to be arbitrarily defined. Sensitive to the scale (the unit) of the predictors
Bayes factor (vs. 0)	The degree by which the probability mass has shifted away from or toward the null value, after observing the data	Ratio of the density of the null value between the posterior and the prior distributions	An unbounded continuous measure of relative evidence. Allows statistically supporting the null hypothesis	Sensitive to selection of prior distribution shape, location and scale
Bayes factor (vs. ROPE)	The degree by which the probability mass has into or outside of the null interval (ROPE), after observing the data	Ratio of the odds of the posterior vs. the prior distribution falling inside of the range of values defined as the ROPE	An unbounded continuous measure of relative evidence. Allows statistically supporting the null hypothesis. Compared to the BF (vs. 0), evidence is accumulated faster for the null when the null is true	Sensitive to selection of prior distribution shape, location and scale. Additionally, a ROPE range needs to be arbitrarily defined, which is sensitive to the scale (the unit) of the predictors

The results tend to suggest that the investigated indices could be separated into two categories. The first group, including the *pd* and the MAP-based *p*-value, presents similar properties to those of the frequentist *p*-value: they are sensitive only to the amount of evidence for the alternative hypothesis (i.e., when an effect is truly present). In other words, these indices are not able to reflect the amount of evidence in favor of the null hypothesis (Rouder et al., 2009; Rouder and Morey, 2012). A high value suggests that the effect exists, but a low value indicates *uncertainty* regarding its existence (but not certainty that it is non-existent). The second group, including ROPE and Bayes factors, seem sensitive to both presence and absence of effect, accumulating evidence as the sample size increases. However, ROPE seems particularly suited to provide evidence in favor of the null hypothesis. Consistent with this, combining Bayes factors with ROPE (BF vs. ROPE), as compared to Bayes factors against the point-null (BF vs. 0), leads to a higher sensitivity to null-effects (Morey and Rouder, 2011; Rouder and Morey, 2012).

We also showed that besides sharing similar properties, the *pd* has a 1:1 correspondence with the frequentist *p*-value, being its Bayesian equivalent. Bayes factors, however, appear to have a severely non-linear relationship with the frequentist index, which is to be expected from their mathematical definition and their sensitivity when the null hypothesis is true. This in turn can lead to surprising conclusions. For instance, Bayes factors lower than 1, which are considered as providing evidence *against* the presence of an effect, can still correspond to a “significant” frequentist *p*-value (see Figures 3, 4). ROPE indices are more closely related to the *p*-value, as their relationship appears dependent on another factor: the sample size. This suggests that the ROPE encapsulates additional information about the strength of evidence.

What is the point of comparing Bayesian indices with the frequentist *p*-value, especially after having pointed out its many flaws? While this comparison may seem counter-intuitive (as Bayesian thinking is intrinsically different from the frequentist framework), we believe that this juxtaposition is interesting for didactic reasons. The frequentist *p*-value “speaks” to many and can thus be seen as a reference and a way to facilitate the shift toward the Bayesian framework. Thus, pragmatically documenting such bridges can only foster the understanding of the methodological issues that our field is facing, and in turn act against dogmatic adherence to a framework. This does not preclude, however, that a change in the general paradigm of significance seeking and “p-hacking” is necessary, and that Bayesian indices are fundamentally different from the frequentist *p*-value, rather than mere approximations or equivalents.

Critically, while the purpose of these indices was solely referred to as *significance* until now, we would like to emphasize the nuanced perspective of existence-significance testing as a dual-framework for parameter description and interpretation. The idea supported here is that there is a conceptual and practical distinction, and possible dissociation to be made, between an effect’s existence *and* its significance. In this context, *existence* is simply defined as the consistency of an effect in one particular direction (i.e., positive or negative), without any assumptions or conclusions as to its size, importance, relevance or meaning. It is an objective feature of an estimate (tied to its uncertainty). On the other hand, *significance* would be here re-framed following its original literally definition such as “being worthy of attention” or “importance.” An effect can be considered significant if its magnitude is higher than some given threshold. This aspect can be explored, to a certain extent, in an objective way with the concept of *practical equivalence* (Kruschke, 2014; Lakens, 2017; Lakens et al., 2018), which suggests the use of a range of values assimilated to the absence of an effect (ROPE). If the effect falls within this range, it is considered to be non-significant *for practical reasons*: the magnitude of the effect is likely to be too small to be of high importance in real-world scenarios or applications. Nevertheless, *significance* also withholds a more subjective aspect, corresponding to its contextual meaningfulness and relevance. This, however, is usually dependent on the literature, priors, novelty, context or field, and thus cannot be objectively or neutrally assessed using a statistical index alone.

While indices of existence and significance can be numerically related (as shown in our results), the former is conceptually independent from the latter. For example, an effect for which the whole posterior distribution is concentrated within the [0.0001, 0.0002] range would be considered to be positive with a high level of certainty (and thus, *existing* in that direction), but also not significant (i.e., too small to be of any practical relevance). Acknowledging the distinction and complementary nature of these two aspects can in turn enrich the information and usefulness of the results reported in psychological science (for practical reasons, the implementation of this dual-framework of existence-significance testing is made straightforward through the *bayestestR* open-source package for R; Makowski et al., 2019). In this context, the *pd* and the MAP-based *p*-value appear as indices of effect existence, mostly sensitive to the certainty related to the direction of the effect. ROPE-based indices and Bayes factors are indices of effect significance, related to the magnitude and the amount of evidence in favor of it (see also a similar discussion of statistical significance vs. effect size in the frequentist framework; e.g., Cohen, 1994).

The inherent subjectivity related to the assessment of significance is one of the practical limitations of ROPE-based indices (despite being, conceptually, an asset, allowing for contextual nuance in the interpretation), as they require an explicit definition of the non-significant range (the ROPE). Although default values have been reported in the literature (for instance, half of a “negligible” effect size reference value; Kruschke, 2014), it is critical to reproducibility and transparency that the researcher’s choice is explicitly stated (and, if possible, justified). Beyond being arbitrary, this range also has hard limits (for instance, contrary to a value of 0.0499, a value of 0.0501 would be considered non-negligible if the range ends at 0.05). This reinforces a categorical and clustered perspective of what is by essence a continuous space of possibilities. Importantly, as this range is fixed to the scale of the response (it is expressed in the unit of the response), ROPE indices are sensitive to changes in the scale of the predictors. For instance, negligible results may change into non-negligible results when predictors are scaled up (e.g., reaction times expressed in seconds instead of milliseconds), which one inattentive or malicious researcher could misleadingly present as “significant” (note that indices of existence, such as the *pd*, would not be affected by this). Finally, the ROPE definition is also dependent on the model type, and selecting a consistent or homogeneous range for all the families of models is not straightforward. This can make comparisons between model types difficult, and an additional burden when interpreting ROPE-based indices. In summary, while a well-defined ROPE can be a powerful tool to give a different and new perspective, it also requires extra caution on the paets of authors and readers.

As for the difference between ROPE (95%) and ROPE (full), we suggest reporting the latter (i.e., the percentage of the whole posterior distribution that falls within the ROPE instead of a given proportion of CI). This bypasses the use of another arbitrary range (95%) and appears to be more sensitive to delineate highly significant effects). Critically, rather than using the percentage in ROPE as a dichotomous, all-or-nothing decision criterion, such as suggested by the original equivalence test (Kruschke, 2014), we recommend using the percentage as a continuous index of significance (with explicitly specified cut-off points if categorization is needed, for instance 5% for significance and 95% for non-significance).

Our results underline the Bayes factor as an interesting index, able to provide evidence in favor or against the presence of an effect. Moreover, its easy interpretation in terms of odds in favor or against one hypothesis or another makes it a compelling index for communication. Nevertheless, one of the main critiques of Bayes factors is its sensitivity to priors (shown in our results here through its sensitivity to model types, as priors’ odds for logistic and linear models are different). Moreover, while the BF appears even better when compared with a ROPE than when compared with a point-null, it also carries all the limitations related to ROPE specification mentioned above. Thus, we recommend using Bayes factors (preferentially vs. a ROPE) if the user has explicitly specified (and has a rationale for) informative priors (often called “subjective” priors; Wagenmakers, 2007). In the end, there is a relative proximity between Bayes factors (vs. ROPE) and the percentage in ROPE (full), consistent with their mathematical relationship.

Being quite different from the Bayes factor and ROPE indices, the Probability of Direction (*pd*) is an index of effect existence representing the certainty with which an effect goes in a particular direction (i.e., is positive or negative). Beyond its simplicity of interpretation, understanding and computation, this index also presents other interesting properties. It is independent from the model, i.e., it is solely based on the posterior distributions and does not require any additional information from the data or the model. Contrary to ROPE-based indices, it is robust to the scale of both the response variable and the predictors. Nevertheless, this index also presents some limitations. Most importantly, the *pd* is not relevant for assessing the size or importance of an effect and is not able to provide information *in favor* of the null hypothesis. In other words, a high *pd* suggests the presence of an effect but a small *pd* does not give us any information about how plausible the null hypothesis is, suggesting that this index can only be used to eventually reject the null hypothesis (which is consistent with the interpretation of the frequentist *p*-value). In contrast, BFs (and to some extent the percentage in ROPE) increase or decrease as the evidence becomes stronger (more data points), in both directions.

Much of the strengths of the *pd* also apply to the MAP-based *p*-value. Although possibly showing some superiority in terms of sensitivity as compared to it, it also presents an important limitation. Indeed, the MAP is mathematically dependent on the density at 0 and at the mode. However, the density estimation of a continuous distribution is a statistical problem on its own and many different methods exist. It is possible that changing the density estimation may impact the MAP-based *p*-value, with unknown results. The *pd*, however, has a linear relationship with the frequentist *p*-value, which is in our opinion an asset.

After all the criticism regarding the frequentist *p*-value, it may appear contradictory to suggest the usage of its Bayesian empirical equivalent. The subtler perspective that we support is that the *p*-value is not an intrinsically bad, or wrong, index. Instead, it is its misuse, misunderstanding and misinterpretation that fuels the decay of the situation into the crisis. Interestingly, the proximity between the *pd* and the *p*-value follows the original definition of the latter (Fisher, 1925) as an index of effect existence *rather than* significance (as in “worth of interest”; Cohen, 1994). Addressing this confusion, the Bayesian equivalent has an intuitive meaning and interpretation, contributing to making more obvious the fact that all thresholds and heuristics are arbitrary. In summary, the mathematical and interpretative transparency of the *pd*, and its conceptualization as an index of effect existence, offer valuable insight into the characterization of Bayesian results, and its practical proximity with the frequentist *p*-value makes it a perfect metric to ease the transition of psychological research into the adoption of the Bayesian framework.

Our study has some limitations. First, our simulations were based on simple linear and logistic regression models. Although these models are widespread, the behavior of the presented indices for other model families or types, such as count models or mixed effects models, still needs to be explored. Furthermore, we only tested continuous predictors. The indices may behave differently when varying the type of predictor (binary, ordinal) as well. Finally, we limited our simulations to small sample sizes, for the reason that data is particularly noisy in small samples, and experiments in psychology often include only a limited number of subjects. However, it is possible that the indices converge (or diverge) for larger samples. Importantly, before being able to draw a definitive conclusion about the qualities of these indices, further studies should investigate the robustness of these indices to sampling characteristics (e.g., sampling algorithm, number of iterations, chains, warm-up) and the impact of prior specification (Kass and Raftery, 1995; Vanpaemel, 2010; Kruschke, 2011), all of which are important parameters of Bayesian statistics.

## Reporting Guidelines

How can the current observations be used to improve statistical good practices in psychological science? Based on the present comparison, we can start outlining the following guidelines. As *existence* and *significance* are complementary perspectives, we suggest using at minimum one index of each category. As an objective index of effect existence, the *pd* should be reported, for its simplicity of interpretation, its robustness and its numeric proximity to the well-known frequentist *p*-value; As an index of significance either the *BF* (vs. *ROPE*) or the *ROPE (full)* should be reported, for their ability to discriminate between presence and absence of effect (De Santis, 2007) and the information they provide related to evidence of the size of the effect. Selection between the *BF* (vs. *ROPE*) or the *ROPE (full)* should depend on the informativeness of the priors used – when uninformative priors are used, and there is little prior knowledge regarding the expected size of the effect, the *ROPE (full)* should be reported as it reflects only the posterior distribution and is not sensitive to the width of a wide-range of prior scales (Rouder et al., 2018). On the other hand, in cases where informed priors are used, reflecting prior knowledge regarding the expected size of the effect, *BF* (vs. *ROPE*) should be used.

Defining appropriate heuristics to aid in interpretation is beyond the scope of this paper, as it would require testing them on more natural datasets. Nevertheless, if we take the frequentist framework and the existing literature as a reference point, it seems that 95, 97, and 99% may be relevant reference points (i.e., easy-to-remember values) for the *pd*. A concise, standardized, reference template sentence to describe the parameter of a model including an index of point-estimate, uncertainty, existence, significance and effect size (Cohen, 1988) could be, in the case of *pd* and *BF*:

“There is moderate evidence (*BF*_ROPE_ = 3.44) [*BF* (vs. *ROPE*)] in favor of the presence of effect of X, which has a probability of 98.14% [*pd*] of being negative (Median = −5.04, 89%CI[−8.31,0.12]), and can be considered to be small (Std. Median=−0.29) [*standardized coefficient*].”

And if the user decides to use the percentage in ROPE instead of the *BF*:

“The effect of X has a probability of 98.14% [*pd*] of being negative (Median=−5.04, 89%*CI*[−8.31,0.12]), and can be considered to be small (Std.Median=−0.29) [*standardized coefficient*] and significant (0.82%*inROPE*) [*ROPE (full)*].”

## Data Availability Statement

The full R code used for data generation, data processing, figures creation, and manuscript compiling is available on GitHub at https://github.com/easystats/easystats/tree/master/publications/makowski_2019_bayesian.

## Author Contributions

DM conceived and coordinated the study. DM, MB-S, and DL participated in the study design, statistical analysis, data interpretation, and manuscript drafting. DL supervised the manuscript drafting. SC performed a critical review of the manuscript, assisted with the manuscript drafting, and provided funding for publication. All authors read and approved the final manuscript.

## Conflict of Interest

The authors declare that the research was conducted in the absence of any commercial or financial relationships that could be construed as a potential conflict of interest.

## Footnotes

1 https://github.com/easystats/easystats/tree/master/publications/makowski_2019_bayesian/data
